# Rural community peer partnerships for improving methamphetamine -associated heart failure screening and engagement in cardiology care (PEER-Heart): Study protocol^☆^

**DOI:** 10.1016/j.dadr.2026.100411

**Published:** 2026-01-29

**Authors:** Maria Alias-Ferri, Cooper B. Kersey, Evan F. Shalen, Ryan Cook, Devin Gregoire, Kim Hoffman, Michelle Beam, Ximena A. Levander, Kellie Pertl, Alexis Stensby, Paul Gonzales, Shanna Smith, Tabetha Evernden, Chris T. Longenecker, P. Todd Korthuis, Brian Chan

**Affiliations:** a Division of General Internal Medicine & Geriatrics, Section of Addiction Medicine, Oregon Health & Science University, Portland, OR, USA; b University of Washington School of Medicine, Seattle, WA, USA; c Department of Medicine, Division of Cardiovascular Medicine, Oregon Health & Science University, Portland, OR, USA; d Saint Louis University School of Medicine, Saint Louis, MO 63104, USA; e Bay Area First Step, USA

**Keywords:** Methamphetamine use disorder, Methamphetamine-associated heart failure, Heart failure screening, Telemedicine, Peer recovery support specialists

## Abstract

**Background:**

Methamphetamine-associated heart failure (MAHF) is increasingly prevalent in rural communities, where limited specialty care and barriers to healthcare engagement hinder early diagnosis and treatment. Peer-led screening with brain natriuretic peptide (BNP) testing, supported by telemedicine, may enhance early detection and linkage to cardiology care.

**Aim:**

PEER-Heart is a hybrid type 1 effectiveness-implementation trial to evaluate the feasibility, acceptability, and effectiveness of a peer-assisted point-of-care screening protocol and telecardiology intervention for MAHF in rural Oregon.

**Methods:**

We will recruit 122 adults reporting methamphetamine use within the past 30 days from two rural Oregon counties. Individuals will be screened for MAHF by peers using a symptom questionnaire, brain natriuretic peptide (BNP) testing, and a mobile electrocardiogram. Individuals who screen positive will be randomized to a peer-facilitated telecardiology intervention or enhanced usual care (EUC). Primary outcome is linkage to heart failure treatment at 2 months. Secondary outcomes include changes in symptom severity, knowledge, and engagement in guideline-directed medical therapy. Implementation barriers and facilitators will be assessed through interviews and focus groups using thematic analysis and the Reach, Effectiveness, Adoption, Implementation, Maintenance (RE-AIM) framework. We hypothesize that peer-assisted telecardiology will result in higher linkage to care. The study will assess the feasibility and acceptability of peer-delivered cardiovascular screening and telecardiology in high-risk populations.

**Conclusion:**

PEER-Heart addresses a critical gap in early detection and management of heart failure for people who use methamphetamine in rural settings. Findings will inform efforts to scale peer-integrated telemedicine programs for underserved populations with complex needs.

## Highlights

• Methamphetamine use is associated with heart failure.
• Peer support and telecardiology improve early detection and care linkage.
• Trial tests feasibility, acceptability, and effectiveness of peer assisted approach.
• Implementation outcomes assessed using qualitative and mixed methods.
• Model addresses stigma and structural barriers to reduce heart disease disparities.

## 1 Background

Methamphetamine use (MAU) has significantly increased in recent years, with the National Survey on Drug Use and Health (NSDUH) reporting a 2.5-fold rise in monthly use among individuals aged 12 or older between 2016 and 2022 (Center for Behavioral Health Statistics, 2017, Center for Behavioral Health Statistics, 2023). Concurrently, treatment rates for MAU doubled over the past decade, making it the third most common reason for substance use disorder treatment after alcohol and opioids (Substance Abuse and Mental Health Services Administration, 2022, Substance Abuse and Mental Health Services Administration, 2023). Rural communities are especially vulnerable due to significant barriers in accessing treatment for substance use disorders and overdose risk (Button et al., 2023).

There is strong evidence linking MAU and the development of heart failure (Manja et al., 2023, Schwarzbach et al., 2020, Somma et al., 2023). Individuals with methamphetamine-associated heart failure (MAHF) tend to develop congestive heart failure with dilated cardiomyopathy resulting in longer hospitalizations, more readmissions (Manja et al., 2023), higher rates of mortality (Davis et al., 2023), worse prognosis and less adherence to guideline-directed medical therapies (GDMT) (Breier et al., 2023) than those with non-MAHF.

Heart failure rates remain significantly higher among individuals who use methamphetamine, even after controlling for other cardiovascular risk factors, and the prevalence of MAHF has increased in recent years, particularly in the Western United States, which is disproportionately affected (Dickson et al., 2021, Manja et al., 2023, Richards et al., 2018, Somma et al., 2023, Al-Yafeai et al., 2024). In this region, MAHF hospitalization rates have climbed to 47 cases per 1000 people, significantly higher than the national average of 7 per 1000 (Dickson et al., 2021). The states of Oregon, Washington, and Nevada also report some of the highest rates of methamphetamine-related emergency department visits (Substance Abuse and Mental Health Services Administration, 2019).

Interventions to identify and connect people at risk of MAHF to healthcare are needed. Screening with brain natriuretic peptide (BNP) along with collaborative care for patients with cardiovascular risk factors can lower the risk of heart failure by detecting issues like left ventricular and diastolic dysfunction before symptoms appear (Ledwidge et al., 2013). BNP is released in response to myocardial stretch and volume overload, making it a sensitive biomarker for early detection of cardiac dysfunction and referral guidance (Gordon and Rempher, 2003). Although MAHF is most commonly described as a dilated cardiomyopathy, some individuals identified through screening may present with other heart failure phenotypes, such as heart failure with preserved ejection fraction or hypertensive heart disease. To our knowledge, there is no evidence that BNP thresholds for heart failure diagnosis differ in patients with methamphetamine-associated cardiomyopathy or MAHF defined using broader criteria. QRS duration (≥120 ms) reflects ventricular conduction abnormalities associated with myocardial remodeling and cardiomyopathy and is common in heart failure populations. QRS prolongation is associated with impaired ventricular function and poorer outcomes, making it a useful, low-cost screening marker to identify individuals who may benefit from further cardiac evaluation (Kashani and Barold, 2005). Collaborative care (Harris et al., 2021) in this context refers to a team-based approach that integrates peer support, primary care, and telecardiology, ensuring patients receive care across settings. This approach is rated 2a recommendation in the 2022 American Heart Association (AHA)/American College of Cardiology (ACC)/Heart Failure Society of America (HFSA) heart failure guidelines (Heidenreich et al., 2022). However, several key barriers impede implementation of screening for people at risk for MAHF. First, people who use methamphetamine often reside in rural settings, where access to medical care, especially specialty cardiology care, is limited (Winstanley et al., 2024). Second, people who use methamphetamine often experience stigma in traditional healthcare settings leading them to avoid care (Forchuk et al., 2024).

To improve healthcare access in rural areas, telemedicine provides a key strategy to help bridge the gap caused by distance (Anawade et al., 2024, Dorsey and Topol, 2020). Telemedicine approaches have improved delivery of drug use, HIV, and hepatitis C treatment (Lin et al., 2019, McKellar et al., 2024, Talal et al., 2022, Talal et al., 2023, Talal et al., 2023, Villanueva-Blasco et al., 2024). Telecardiology is a feasible option for heart failure care, (Yamano et al., 2022) with strategies to prevent hospitalizations and optimize therapy based on remote monitoring, enhancing patient outcomes in underserved regions (Pegoraro et al., 2023).

To address barriers to engaging in healthcare, integrating non-clinician professionals has proven effective for treating various diseases (Kim et al., 2024). For example, peer recovery support specialists (peers) are individuals with lived experience of substance use and recovery who help others engage in substance use prevention and treatment (Substance Abuse and Mental Health Services Administration (SAMHSA, 2024). Peers provide experiential credentials, offering a unique avenue to engage and retain non-treatment-seeking people who use drugs (PWUD). Integrating peers with telecardiology may improve accessibility, acceptability, and support for engaging in healthcare treatment for PWUD. PEER-Heart is a community engaged feasibility and acceptability implementation study that aims to fill a critical gap in the early detection and treatment of MAHF by identifying individuals at risk and connecting them to peer-assisted telecardiology care. We hypothesize that peer-assisted telecardiology increases linkage to care compared to peer assisted EUC and will assess contextual determinants influencing adoption and sustainability in rural healthcare.

## 2 Methods

We report the PEER-Heart protocol (Version 2 – 04/05/2024) in concordance with the 2013 SPIRIT guidelines (Chan et al., 2013) (Supplementary 1).

### 2.1 Overview of the study design

The PEER-Heart study is a hybrid type 1 effectiveness-implementation trial (Curran et al., 2012), which simultaneously evaluates the clinical effectiveness of an intervention while gathering data on implementation processes and contextual factors to inform future scale-up. This dual focus allows researchers to explore not only the peer-facilitated telecardiology intervention compared with enhanced usual care but also factors that influence its implementation among people who use methamphetamine in rural areas. The study aims to:

• Aim 1. Adapt and implement a peer-directed MAHF screening program and assess its feasibility and acceptability for people with MAU living in rural communities. The study will assess how well peers are able to conduct the screening, identify barriers and facilitators to implementation, and examine disparities in reach across demographic groups**.**
• Aim 2. Determine feasibility, acceptability, and effectiveness of a novel peer-assisted telecardiology program to screen and engage patients at risk for MAHF in cardiology care at 2 months. This study is a non-blinded, two-arm, randomized controlled trial to test the superiority of peer-assisted telecardiology in comparison to enhanced usual care (EUC) for heart failure treatment in people who use methamphetamine in rural areas of Oregon (clinicaltrials.gov NCT06461962).
• Aim 3. To identify and characterize facilitators and barriers to implementation. Using individual interviews and focus groups during Aim 1 study activities, we seek to identify and understand strategies for scaling and sustaining the PEER-Heart model in other settings.

### 2.2 Study setting

This study takes place in two rural counties, Coos and Curry, on the Southern Oregon Coast as a collaboration between local community partner Bay Area First Step (BAFS) and Oregon Health & Science University (OHSU) in Portland, Oregon. These counties are highly rural, with more than 98 % and 100 % of their territory, respectively, classified as rural (Census.gov | U.S. Census Bureau Homepage, 2025). Coos County has a population density of ~39 persons/sq mi, while Curry County is even more sparsely populated at ~13 persons/sq mi (Census.gov | U.S. Census Bureau Homepage, 2025). These characteristics contribute to limited healthcare infrastructure and access in both areas.

BAFS is a peer-led community-based service organization that provides housing and peer-delivered services designed to support PWUD and their families in these rural counties. BAFS leadership and staff contributed to the design of the intervention providing input on study procedures, recruitment strategies and feasibility within the local service context. BAFS plays an active role in the community and fosters strong relationships with potential study participants. In the past 12 months, BAFS has served over 1500 individuals, the majority of whom (88 %) were between the ages of 25 and 64. Most clients identified as White (74 %) and male (59 %), although BAFS also reaches a diverse population. BAFS has prior experience implementing and recruiting participants for new service and research initiatives, supporting the feasibility of achieving the proposed sample size. BAFS peers will recruit, screen, and link eligible individuals to care. Two BAFS peers have been trained and serve as research assistants, collecting participant consent and survey data.

In Oregon, to be certified as a peer, individuals must be older than 18 years, successfully complete an Oregon Health Authority approved training program and pass a criminal history background check. Depending on the specialization, training is 40 h for Peer Support Specialists (PSS), or 80 for Peer Wellness Specialists (PWS) and includes essential skills for peer support, person-centered communication techniques, and a range of other key competencies and guiding principles. Oregon requires recertification every three years and peers must complete a minimum number of hours, depending on the certification, of continuing education.

### 2.3 Study participants

The PEER-Heart study recruits individuals who are residents of Coos and Curry Counties, Oregon—the BAFS service area. As this is a feasibility study conducted within existing community-based services, participants will be recruited without predefined targets for gender or race/ethnicity, and enrollment is expected to reflect the demographics of individuals accessing participating sites.

Individuals meet the following inclusion criteria: age 18 years or older, active health insurance, self-report of any MAU in last 30 days and regular use over the prior year, no client-reported diagnosis of heart failure, and able to communicate in English. We exclude individuals who report existing engagement in medical care for heart failure and/or are actively taking medications specifically prescribed for the treatment of heart failure. The study excludes prisoners and detained individuals.

### 2.4 Sample size and power

As part of Aim 1 and to reach enrollment goals, we plan to screen at least 220 individuals for elevated BNP levels, identifying people who use methamphetamine at risk for heart failure. This estimate reflects an initial screening target; however, the study is powered on the number of individuals who screen positive rather than the total number screened, and screening will continue as needed to enroll 122 participants with elevated BNP. We will continue screening as needed to reach our target enrollment. No prior study has reported the prevalence of positive screening (using BNP) for heart failure in community-dwelling people who use methamphetamine who are not already diagnosed with cardiomyopathy, however one prior cohort study with people who use methamphetamine presenting to the emergency department found that 63 % had abnormal BNP levels (Richards et al., 2018), which likely overestimates the prevalence in a community-dwelling population. In contrast, in the STOP-HF randomized controlled trial, approximately 40 % of community-dwelling primary care patients with cardiovascular risk factors had a BNP > 50 (Ledwidge et al., 2013), providing a more conservative reference point for BNP positivity in non-acute settings. Based on these findings, we anticipate that 122 individuals from those will have elevated BNP levels indicative of possible heart failure and meet other eligibility criteria, making them eligible for enrollment in the Aim 2 randomized controlled trial. Assuming a 10 % linkage to cardiology care in the enhanced usual care (EUC) arm, a sample size of 122 participants allows us to detect a clinically meaningful difference in linkage rates between study arms with adequate statistical power. So based on these considerations, this sample sizeallows detection of a 20 % increase in the linkage to cardiology care in the telecardiology arm (i.e., achieving a rate of 30 % at 2 months), assuming 80 % power for a two-sided test of proportions at α= 0.05. This baseline estimate is informed by linkage rates reported in symptomatic, hospitalized populations, which may differ significantly from outpatient screening populations, in whom follow-up care rates are expected to be lower.

### 2.5 Participant screening procedures

BAFS Peers present study information to potential participants through their usual outreach and engagement protocols that support PWUD. Recruitment may occur in parks, community gatherings, recovery housing, and among people presenting for care in local healthcare facilities or to BAFS for treatment assessment. We will post flyers in community-based settings, recovery housing, and local healthcare facilities including BAFS services intake site.

Peers will screen individuals at risk for MAHF and those who screen positive and provide informed consent will be enrolled as study participants. Pre-screening takes approximately 10 min and consists of a heart failure symptom questionnaire (administered and collected via REDCap), a point of care (POC) testing which includes B type natriuretic peptide collected via fingerstick (BNP; Abbott i-STAT) and mobile electrocardiogram (ECG; KardiaMobile®) (Appendix 1). While BNP is highly sensitive, its specificity can be enhanced by combining it with QRS duration on electrocardiogram ECG (Krüger et al., 2004). A positive screen for possible MAHF is defined as BNP ≥ 50 pg/ml, or QRS width ≥ 120 ms, or presence of heart failure symptoms.

Positive screening findings may be driven by elevated BNP, prolonged QRS duration, or self-reported symptoms, which can be non-specific and attributable to conditions other than heart failure, and are intended to identify individuals who may benefit from further clinical evaluation rather than establish a diagnosis.

### 2.6 Study assessments

**Fig. 1:**
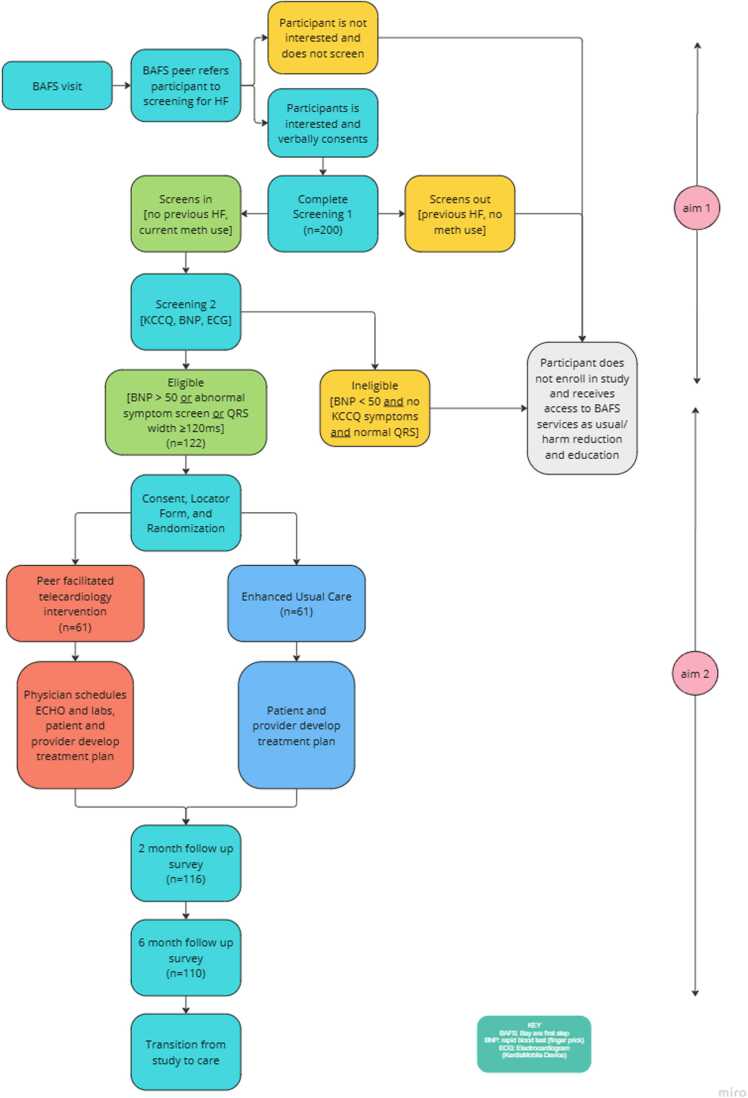
Overall trial design and procedures for the PEER-Heart study.

Eligible participants identified through the screening process will complete follow-up surveys at 2 and 6 months (Fig. 1). Surveys are collected using REDCap (Table 1), in-person or by phone, including: demographics and housing; access to medical care and prescribed medications; heart failure symptom severity (adapted from the Kansas City Cardiomyopathy Questionnaire); heart failure diagnosis and cardiology treatment; drug and alcohol use patterns; addiction treatment utilization; harm reduction services; overdose history and related complications; perceived stigma; peer support services; quality of life (including a 0–10 visual analog scale); experiences with telehealth services. Participants in the intervention arm will additionally complete items assessing satisfaction with peer-delivered telecardiology support as part of feasibility and acceptability assessments (Weiner et al., 2017).

**Table 1:** Summary of Measures Included in the 2 and 6-Month Follow-Up Survey.

**Domain**	**Instrument**	**Description**	**Number of Items**	**Response Format**
**Demographics and Housing**	Study-specific	Recent homelessness, primary sleeping location	2	Dichotomized Open-ended
**Access to Medical Care and Prescribed Medications**	Study-specific	Access to primary care, barriers, site of care, transportation; prescribed medications	8	Dichotomized Checklist Multiple choice
**Heart Failure Symptom Severity**	Kansas City Cardiomyopathy Questionnaire (KCCQ-12, adapted)	Frequency and impact of HF symptoms (fatigue, swelling, shortness of breath, orthopnea)	4	Likert
**Heart Failure Diagnosis and Treatment**	Study-specific	HF diagnosis, echocardiogram, appointments, medication adherence, hospital visits	7	Dichotomized Checklist Visual analog scale
**Drug and Alcohol Use Patterns**	Study-specific, ASI-Lite	Types of substances used, frequency, route of administration, craving, alcohol use	10	Checklist # of days Sliding scale
**Addiction Treatment Utilization**	Study-specific	Inpatient/outpatient treatment, self-help groups, addiction medications, contingency management	9	Dichotomized Open-ended Checklist
**Harm Reduction Services**	Study-specific	Injection practices, equipment sharing, source of syringes and supplies	7	Multiple choice # of times Checklist
**Overdose History and Related Complications**	Study-specific	Overdose events, naloxone administration, stimulant-related physical or mental complications	5	Dichotomized Open-ended # of times
**Perceived Stigma**	Substance Use Stigma Mechanisms Scale (adapted)	Internalized and anticipated stigma related to drug use	7	Likert Dichotomized
**Peer Support Services**	Peer Support Scale (PSS-10, adapted)	Quality, respect, safety, sensitivity, and perceived benefit of peer support received	10	Dichotomized Likert
**Quality of Life (including a 0–10 visual analog scale)**	CDC HRQoL + Visual Analog Scale	Self-rated well-being, general health, days with poor physical/mental health and functional limitations	5	0–10 scale Multiple choice # of days
**Experiences with Telehealth Services**	Study-specific	Satisfaction, usability, comfort, willingness to use again	8	Dichotomized Likert
**Feasibility and Acceptability Assessments*****(intervention arm only)***	Weiner et al., 2017 – AIM (Acceptability of Intervention Measure)	Acceptability of peer-delivered telecardiology support: approval, appeal, liking, welcome	4	Likert

Management of identified cardiovascular disease will be based on clinical practice routine and to the discretion of treating clinicians. Time to diagnostic evaluation, including echocardiography, may vary across participants and study arms. Participants in the EUC arm receive care consistently with local standard practices, and equipoise is maintained because local providers may navigate logistical or system-level hurdles as efficiently as the telecardiology team. The study does not restrict participants’ access to any diagnostic testing or treatment.

### 2.7 Description of the PEER-Heart intervention

Individuals who screen positive and provide consent to enroll will be randomized to peer-assisted telehealth-facilitated intervention (intervention condition) or peer-assisted EUC (control condition). In both study arms, peers facilitate engagement to care.

#### 2.7.1 Telecardiology intervention group

Participants will receive care remotely (via telephone visits or internet/EPIC MyChart facilitated virtual visits) with the telehealth-cardiology team. This team consists of a cardiologist and a clinical coordinator based out of the states alone urban academic medical center (OHSU). Participants randomized to the peer-assisted telehealth-cardiology intervention arm will receive referrals for local echocardiogram and lab testing, a virtual consultation with a cardiologist, and transitional care coordinated by a nurse care manager and peer. The cardiologist will initiate GDMT for those with reduced left ventricular ejection fraction, provide diuretics as needed for decongestion, and may recommend additional diagnostic testing. The telecardiology intervention will provide care for up to 3-months, then arrange referral with a local cardiovascular specialist. Follow-ups will occur at least monthly or as needed, and patients stable without heart failure will transition to primary care. Peers will facilitate the engagement of participants with tele-cardiology care intervention, and support participants in accessing labs, imaging, and telehealth resources.

#### 2.7.2 Enhanced usual care group

Participants randomized to EUC arm will receive a peer-assisted referral to a local primary care provider for the management of heart failure. Follow-up care will be based on current local practices. Like the intervention arm, peers facilitate initial engagement of participants with local treatment providers.

### 2.8 Outcomes

#### 2.8.1 Primary outcome: linkage to care at 2-months

The primary outcome is linkage to follow-up care at two months post-enrollment, defined as having successfully completed a visit with a cardiologist for evaluation of risk related to methamphetamine-associated heart failure. We hypothesize that the intervention group will experience a higher proportion of linkage to cardiology care at two months post-randomization compared to the usual care group. The sample size determination is based on detecting a meaningful difference in this outcome (detailed in the *2.4. Sample Size and Power* section).

#### 2.8.2 Secondary outcomes

Secondary outcomes include participants’ reported changes in confidence and knowledge of MAHF-associated heart failure risk, severity of heart failure symptoms, and adherence to GDMT.

#### 2.8.3 Implementation assessment

Additionally, the study will assess implementation outcomes such as the feasibility and acceptability of peer-assisted MAHF screening, barriers and facilitators to implementing telecardiology care, and drivers of intervention effectiveness, maintenance, and sustainability using the RE-AIM (reach effectiveness adoption implementation maintenance) framework.

We will evaluate implementation and barriers by conducting focus groups consisting of 15 peers from participating community-based organizations and clinicians both involved and not involved in the project during the first and third year of the study, exploring formative feedback and tele-cardiology intervention factors. Additionally, individual qualitative interviews will be conducted with 15 participants in each group (total N = 30) to explore the screening intervention and linkage to cardiology care within the client-peer relationship. All interviews and focus groups will take place in convenient, private, and safe settings, either in-person or virtually.

To carry out the analysis the team will employ thematic analysis to create a coding scheme, practice coding, and revise iteratively, following common qualitative study practices (Braun and Clarke, 2006). Qualitative analysts will complete line-by-line coding of five sample transcripts independently, discuss transcript coding, reach a consensus about code definitions, and finalize a codebook (of all codes and their definitions). We will review a sample of transcripts to ensure reliable coding, assess the need for new codes, or revise existing code definitions. We will hold weekly meetings to discuss the coding process, resolve problems, ensure consistency, and adjust the interview guide or sample as needed. The team will read for negative cases (i.e., cases that do not fit themes) and will conduct a member check at each site. We will use qualitative analysis software (ATLAS.ti) for data management and analysis.

### 2.9 Analysis

#### 2.9.1 Aim 1

We assess acceptability by determining what proportion of peers can conduct the screening outreach intervention; we will assess fidelity based on which aspects of screening were completed. During audit and feedback meetings with peers, we will assess ease and barriers to implementing the screening as intended. We will descriptively examine patterns of reach and acceptability within the rural population served.

As part of the implementation evaluation of Aim 1, clinic study investigators will review positive screens and subsequent testing results (i.e., echocardiography has been performed, and whether the echocardiogram reveals structural heart disease).

#### 2.9.2 Aim 2

We hypothesize that participants assigned to the intervention group will experience a higher proportion of linkage to cardiology care at 2-months (primary outcome). We will test statistical imbalance between the intervention and control groups on baseline demographic characteristics; those showing significant imbalance will be included as covariates in the primary outcome model. Linkage to cardiology care at 2-months, the primary outcome, will be analyzed with a generalized estimating equation (GEE) model with binomial family and logit link, which allows us to control correlation introduced by peer site. Missing data is imputed as not retained, and robust standard errors will be used for hypothesis tests.

Secondary outcomes will be tested using GEE models with appropriate distributions and link functions; we test hypotheses of within- and between-group changes over time (screening, 2, and 6 months), comparing the intervention to EUC. Missing secondary outcome data will be handled using multiple imputations if missing at random is a reasonable assumption, single imputation if missing not at random. Sensitivity analyses will be carried out to evaluate the impact of missing data.

Details on the sample size justification, including assumptions about baseline rates and detectable effect sizes, are provided in Section 2.4. Sample size and power.

#### 2.9.3 Aim 3

For Aim 3, we will analyze qualitative data from focus groups and individual interviews using thematic analysis to identify key barriers and facilitators to implementing the peer-assisted telecardiology model. Following standard qualitative procedures, including codebook development and iterative coding, this analysis will focus on contextual factors, perceived value of the intervention, and considerations for scale-up and sustainability in rural settings. Particular attention will be paid to implementation strategies, adaptations across sites, and stakeholder-reported challenges and successes. Data will be managed and coded using ATLAS.ti.

### 2.10 Study incentives

Participants will receive up to $120 cash for participating in this study. Anyone who completes the screening assessments will be compensated $5. Those who screen positive and enroll in the study will be compensated $20 for the baseline survey, $30 for the 2-month survey, and $30 for the 6-month survey. Participants invited to participate in the qualitative interviews will receive an additional $40 following the interview. Cash is the preferred and most practical compensation, as few participants have access to transportation to travel to the nearest gift card vendor in this rural area.

### 2.11 Ethics and dissemination

The study has been reviewed and approved by the Oregon Health & Science University IRB (IRB#26150). Participant data will be collected and managed using REDCap, a web-based application designed to support data confidentiality. Participant information will be anonymized and assigned unique study identifiers to protect personal identities and will be de-identified and stored in encrypted databases, accessible only to authorized research personnel. In addition, a Certificate of Confidentiality was obtained to further protect sensitive participant information from compelled disclosure. Any breaches of confidentiality will be reported immediately to the IRB and other appropriate authorities.

## 3 Discussion

This study represents the first effort to assess the feasibility and acceptability of peer-assisted point-of-care screening and linkage to telecardiology for people who use methamphetamine and are at risk of heart failure in a rural setting. By integrating peers into intervention, we aim to overcome key barriers such as stigma and mistrust, providing holistic, trauma-informed, and accessible support while facilitating timely screening and connection to cardiology care (Kane et al., 2024, Spencer et al., 2024, Avalone et al., 2024, Harris et al., 2022). Additionally, by facilitating screening more quickly than conventional medical clinics, this approach allows for expanded reach, linking more individuals to specialized care. Connecting people at risk to MAHF with cardiology specialists via telehealth helps to alleviate the shortage of resources, facilities, and professionals in rural areas. Peer-assisted treatment ensures better patient engagement and provides a sustainable approach to treatment, as peers can guide and assist patients in navigating the healthcare system, which they are often unfamiliar with, ultimately improving the continuity and quality of care (Jack et al., 2018).

Telehealth, particularly telecardiology, offers significant advantages for rural populations with limited access to healthcare resources and specialized professionals. It allows patients to receive timely consultations and follow-up care without the need for long-distance travel, a critical benefit for those in remote areas (Benavides-Vaello et al., 2013). Telehealth has shown promise in managing chronic conditions like heart failure (Mohammadzadeh et al., 2022) and improving access to addiction treatment, as demonstrated by successful programs such as Peer Assisted Telemedicine for Hepatitis C and Syphilis (PATHS) (Spencer et al., 2024). When paired with peer support, as in our study, telecardiology can broaden reach, enhance trust and engagement, improve linkage to cardiology for individuals who might otherwise avoid treatment, addressing acceptability concerns for stigmatized populations (Sakakibara et al., 2019, Su and Yu, 2019). We anticipate that this model will result in a higher proportion of individuals being connected to cardiology services within two months compared to enhanced usual care. Focus on MAHF is especially relevant, as early identification and intervention are critical to improving outcomes in this high-risk population (Breier et al., 2023, Dickson et al., 2021). Peer-assisted telecardiology also presents a scalable and sustainable care model that could be adapted and implemented in other rural or resource-limited settings, potentially transforming access to specialty care for vulnerable populations (Mahalwar et al., 2023, Sakakibara et al., 2019, Su and Yu, 2019).

If effective, the peer-assisted telehealth approach could be expanded to other communities partnering with local peer organizations and scaled to other chronic conditions and rural areas, offering a replicable framework for integrating telehealth with peer support across diverse healthcare landscapes (Ashford et al., 2018, Bazzi et al., 2024, Spencer et al., 2024). Additionally, this model holds potential for reducing healthcare disparities in marginalized populations, while improving access to specialist care and mitigating the impact of geographic and social isolation (Watson et al., 2022). Furthermore, the peer assisted screening protocol for individuals at risk of MAHF could be adapted in urban settings faced with similarly high rates of MAU.

To our knowledge, this is the first study to assess BNP screening for heart failure risk among community-dwelling people who use methamphetamine. Previous studies focused on emergency or inpatient populations, which likely overestimate prevalence. These community-based estimates provide a novel contribution to guide early identification and linkage to cardiology care.

This study has several limitations that may impact its findings and generalizability. Firstly, the lack of a double-blind design could influence participant responses and potentially lead to expectancy bias, affecting the validity of the results. Additionally, the reliance on peer facilitators for screening and intervention may introduce variability in the quality and consistency of care provided, as potential biases could arise from the peer-selection process, with volunteers possibly not representing the broader population.

## 4 Conclusion

The PEER-Heart study addresses a critical gap in the early detection and treatment of methamphetamine-associated heart failure (MAHF) by implementing a novel, scalable model that combines peer-assisted screening with telecardiology. In the context of rising MAU and limited access to specialty care, particularly in rural areas, this intervention seeks to overcome structural barriers such as stigma, transportation, and healthcare shortages. By embedding peers in the care pathway, the study enhances trust, improves participation among people who might otherwise avoid medical services, and facilitates timely linkage to cardiology care. Preliminary findings will inform broader implementation across rural health systems with the aim of improving outcomes and reducing hospitalizations and mortality related to MAHF.

The study contributes a replicable implementation framework for peer-supported telehealth interventions that can be adapted to other chronic conditions and underserved populations. Its emphasis on community engagement, equity, and sustainability offers important guidance for health systems designing care models in low-resource settings. However, successful scale-up will require a responsive care system that ensures fidelity in peer-delivered services and fosters strong collaboration between peers and clinical staff (Scannell, 2021, Scott Kruse et al., 2018). PEER-Heart findings have the potential to shape future telehealth strategies that advance access, equity, and continuity of care in hard-to-reach populations.

## CRediT authorship contribution statement

**Paul Gonzales:** Writing – review & editing, Investigation, Funding acquisition. **Shanna Smith:** Writing – review & editing, Investigation. **Maria Alias-Ferri:** Writing – original draft, Methodology, Investigation. **Kellie Pertl:** Writing – review & editing, Project administration, Methodology, Investigation. **Alexis Stensby:** Writing – review & editing, Investigation. **Korthuis P Todd:** Writing – review & editing, Visualization, Methodology, Funding acquisition. **Ryan Cook:** Writing – review & editing, Methodology, Funding acquisition, Formal analysis, Conceptualization. **Brian Chan:** Writing – review & editing, Visualization, Methodology, Investigation, Funding acquisition, Conceptualization. **Devin Gregoire:** Writing – review & editing, Formal analysis. **Tabetha Evernden:** Writing – review & editing, Methodology, Investigation. **Cooper B. Kersey:** Writing – review & editing, Methodology, Investigation. **Longenecker Chris:** Writing – review & editing, Funding acquisition, Conceptualization. **Evan F. Shalen:** Writing – review & editing, Methodology, Investigation. **Ximena A. Levander:** Writing – review & editing, Methodology, Investigation. **Kim Hoffman:** Writing – review & editing, Investigation, Conceptualization. **Michelle Beam:** Writing – review & editing, Investigation.

## Declaration of Competing Interest

The authors declare the following financial interests/personal relationships which may be considered as potential competing interests: This study is fully granted by the American Heart Association (10.13039/100000968AHA) Rural PRO-CARE network grant (AHA23HERNPRH1150360)
