# Supplementary material for: Rural community peer partnerships for improving methamphetamine -associated heart failure screening and engagement in cardiology care (PEER-Heart): Study protocol
Source: Drug Alcohol Depend Rep. 2026 Jan 29;18:100411. doi: 10.1016/j.dadr.2026.100411 (PMC12887167; doi:10.1016/j.dadr.2026.100411)
Supplement: Supplementary file 1 — Supplementary material [file mmc1.docx]

**Appendix 1 – Study Enrollment Workflow**

## Screening and Eligibility Criteria

1. **Aim 1**
   - Potential participants will be eligible to participate in the study if they meet the following inclusion criteria:
     1. Any methamphetamine use in the past 30 days
     2. Age 18 years or older
     3. No client-reported diagnosis of heart failure
     4. Able to communicate in English
   - Exclusion:
     1. None
2. **Aim 2**
   - Pre-Screening Eligibility Criteria: People who meet Aim 1 eligibility and have a p*ositive* screening for MAHF (defined as BNP ≥50 pg/ml12, *or* QRS is wide, *or* KCCQ score of 13 or less) are eligible to enroll in the study.
   - Aim 2 Exclusion:
     1. Self-reported engagement in medical care for heart failure in past 6 months.
     2. Actively taking medications for heart failure.
3. **Aim 3**
   - Interviews: Community-based organization peers and their client who reports methamphetamine use in the past 30 days are eligible to participate in the dyadic interviews. Clients enrolled in the study will also complete an individual interview.
   - Focus Groups: Peers at participating community-based organizations are eligible for participation.

## Study Enrollment Visit Checklist

Be sure to check that all the following steps have been completed:

| ​☐​ | Created a record for the participant in the Master Enrollment Log in REDCap |
| --- | --- |
| ​​☐​ | Verbally go over the information Sheet (in REDCap) and select yes/no depending on participant willingness to participate |
| ​​☐​ | Completed **screening 1** questions in REDCap |
| ​☐​ | [If eligible:  Patient-defined regular use of methamphetamine in the last year  Any methamphetamine use in the past 30 days  Age 18 years or older  No client-reported diagnosis of heart failure  Able to communicate in English]  CONTINUE |
| ☐​ | Begin BNP Testing and while you wait for results: |
| ​☐​ | Complete **Screening 2** in REDCap |
| ​​☐​ | Conduct ECG |
| ☐​ | Enter results in **ECG and BNP** form in REDCap |
| ☐​ | [if eligible:  BNP ≥50 pg/ml12, *or*  QRS = wide, *or*  KCCQ score of 13 or lower]  CONTINUE |
| ☐​ | Complete **signed consent form** (signed and dated by participant) via REDCap |
| ☐ | Complete **staff attestation** form in REDCap |
| ☐​ | Complete **locator forms** in the Master Enrollment project in REDCap |
| ☐ | Using the module in REDCap assign **Randomization** |
| ☐ | Complete the AIM questionnaire regardless of eligibility |
| ​​☐​ | [depending on randomization] Facilitate connection to TH (OHSU) or TAU (prime + peer hand off) |
| ​​☐​ | Offer OHP assistance (if needed) |
| ☐​ | [If randomized to TH] fill out and send ROI to bay area hospital (see below) |
| ​​☐​ | Schedule next peer visit (see **visit window forms** in REDCap) |
| ☐​ | Pay out cash incentive(s)  **Fill out paper receipts** and report in the **cash log** |
