# Supplementary material for: Rural community peer partnerships for improving methamphetamine -associated heart failure screening and engagement in cardiology care (PEER-Heart): Study protocol
Source: Drug Alcohol Depend Rep. 2026 Jan 29;18:100411. doi: 10.1016/j.dadr.2026.100411 (PMC12887167; doi:10.1016/j.dadr.2026.100411)
Supplement: Supplementary file 4 — Supplementary material [file mmc4.pdf]

Information Sheet & Authorization
Screening

IRB# 26150

TITLE: Rural Community Peer Partnerships for Improving Methamphetamine-Associated Heart Failure Screening and Engagement (PEER-Heart)

CO-PRINCIPAL INVESTIGATORS: Todd Korthuis, MD, MPH (503) 494-6551
Brian Chan, MD, MPH (503) 494-2010

WHO IS PAYING FOR THE STUDY?: The American Heart Association

WHY IS THIS STUDY BEING DONE?:
The overall purpose of this study is to learn more about the use of telemedicine with help from a peer to identify possible heart failure for people who use drugs and to assess linkage to care over time. Telemedicine is a way for healthcare providers to meet with patients remotely, over the phone or a computer. We are hoping to find out better ways to treat people living with both heart failure and a substance use disorder in the community setting.

The purpose of this portion of the study is to screen you for evidence of heart failure by testing your blood and completing an electrocardiogram (ECG). If you agree to the rapid BNP (B type natriuretic peptide) blood test and ECG, then you may be eligible to participate in a study that tests two peer-supported treatment strategies.

You have been invited to be in this research study because you may be at risk for developing heart failure. Screening for heart failure and treating it early may improve health overall. If your screening tests for heart failure are positive, you may be eligible to be treated through telemedicine treatment. The use of telemedicine may help provide access to treatment to those who cannot attend treatment in a doctor's office.

If your test is positive and you want to participate in the full study, you will be provided with more information and will be asked to sign a full study consent form.

We are also asking you to provide de-identified blood and information for a blood/data bank, also called a repository. None of your direct identifying information (like name, email) will be included in this limited dataset but dates of study procedures may be included. These samples will be stored indefinitely and may be used and shared in the future for research.

WHAT TESTS ARE INVOLVED IN THIS STUDY?:
Pre-screening: This portion the study includes completing a brief pre-screening questionnaire to see if you are eligible for screening. We anticipate the pre-screening questionnaire will take approximately 10 minutes to complete.

Screening: This portion of the study includes a rapid BNP test, which involves a finger prick, an ECG, and completing a screening questionnaire. We anticipate this will take approximately 30 minutes to complete. The results of the tests will be available right away. You will have the option of completing the finger prick test on yourself. If you chose to do this, you will be given an instruction sheet. A Bay Area

First Step staff member will be with you to answer questions. You do not have to do the finger prick yourself. If you prefer, a staff member will do the finger prick.

To record an electrocardiogram (ECG) you will be asked to touch your fingers to the metal pads. If you're able, you will also touch a third pad to your left knee or ankle. You will hold this position for approximately 30 seconds during the recording. Try not to move or speak during the recording. You won't feel anything from the device as it does the recording. The ECG will be collected using a device called KardiaMobile. The ECG data will be automatically sent to AliveCor's (the manufacturer of the ECG device) cloud system, where the study team will be able to review the results. We will use a code to identify you so none of your direct identifying information (like name, email) will be included in this data.

If your rapid heart failure test comes back negative, you will be eligible for retesting in 6 months.

If your rapid test is positive and you want to participate in the full study, we will ask you a few additional questions to confirm that you are eligible. These questions are part of the study Inclusion/Exclusion list, and will take about five minutes to complete.

If you have any questions, concerns, or complaints regarding this study now or in the future, or you think you may have been injured or harmed by the study, contact Todd Korthuis (503) 494-6551 or Brian Chan at (503) 494-2010.In the future, your samples and information may be given to researchers for other research studies. The samples and information will be labeled as described in the **WHO WILL SEE MY PERSONAL INFORMATION?** section.

WHAT RISKS CAN I EXPECT FROM TAKING PART IN THIS STUDY?:
Although we have made every effort to protect your identity, there is a minimal risk of loss of confidentiality. To help us protect your privacy, we have obtained a federal Certificate of Confidentiality to protect your privacy even from people who try to get your information using a court order. One exception is if you agree that we can give out research information with your name on it. Another exception is information about child or elder abuse or neglect and harm to yourself or others or communicable disease reporting. This doesn't prevent you from releasing the information yourself.

During the rapid test finger prick, you may feel some pain in your finger. Risk associated with ECG recording includes risk of false positive recording that leads to subsequent testing. There is no substantial risk of direct harm or injury related to ECG recording.

WHAT ARE THE BENEFITS OF TAKING PART IN THIS STUDY?:
You may or may not benefit from being in this study. However, by serving as a subject, you may help us learn how to benefit patients in the future. You may benefit by being diagnosed with heart failure.

WHAT ARE THE ALTERNATIVES TO TAKING PART IN THIS STUDY?:
You may choose not to be in this study.

WILL I RECEIVE RESULTS FROM THIS STUDY?
We will give you the results of your rapid screening test at the time of collection. The results will not be placed in your medical record.

WHO WILL SEE MY PERSONAL INFORMATION?:
In this study we will take steps to keep your personal information confidential, but we cannot guarantee total privacy. However, we will do our best to keep your information confidential by keeping it coded on

an encrypted computer. Locator forms will be kept in locked cabinets that only the researchers have access to. We may request your social security number in order to process any payments for participation.

The investigators, study staff, and others at OHSU may use the information we collect and create about you in order to conduct and oversee this research study and store in a repository for future research. Any samples and information from you that are stored in a research repository will be de-identified. This means the samples and information will not contain any information from or about you that would link them to you. Samples and information stored in the repository includes demographic information, survey responses, substance use information and history, and test results. These samples and information may be released to other investigators for other research studies.

We may have to release this information to others for example, if the study is audited. However, we would try to do so without information that could identify you. This release could be to the Institutional Review Board (ethics review committee) at OHSU, the funder of the study, the FDA or Office of Human Research Protection (agencies that oversee research). Bay Area First Step will release your ECG data to Alivecor and OHSU for the screening activities described above. We may release your information to others outside of OHSU who are also involved in conducting the research, including the University of Washington.

If your information goes outside of OHSU, it might not be protected under federal law from being used or further shared. We would like your permission to keep your contact information and blood sample indefinitely. If you decide you don't want us to use your name and information for this research, you can request this by contacting us at:

Brian Chan, MD, MPH
Department of Internal Medicine
Oregon Health & Sciences University
3181 SW Sam Jackson Park Road, Portland, OR 97239
chanbri@ohsu.edu

Your request will be effective as of the date we receive it. However, health information collected before your request is received may continue to be used and disclosed to the extent that we have already acted based on your authorization.

You do not have to allow the use and disclosure of your health information in the study, but if you do not, you cannot be in the study. If you choose not to participate, or if you decide to stop at any time, that will not affect your ability to receive health care at OHSU or insurance coverage.

WILL ANY OF MY INFORMATION OR SAMPLES FROM THIS STUDY BE USED FOR ANY COMMERCIAL PROFIT?

Samples and information about you or obtained from you in this research may be used for commercial purposes, such as making a discovery that could, in the future, be patented or licensed to a company, which could result in a possible financial benefit to that company, OHSU, and its researchers. There are no plans to pay you if this happens. You will not have any property rights or ownership or financial interest in or arising from products or data that may result from your participation in this study. Further, you will have no responsibility or liability for any use that may be made of your samples or information.

WHAT ARE THE COSTS OF TAKING PART IN THIS STUDY?:

It will not cost you anything to participate in this study. You will receive $5 for completing the pre-screening questoins. If eligible for screening, you will receive an additional $20 for completing rapid BNP

test, ECG, and screening questions.

WHERE CAN I GET MORE INFORMATION?:

This research is being overseen by an Institutional Review Board ("IRB"). You may talk to the IRB at (503) 494-7887 or irb@ohsu.edu if:

- Your questions, concerns, or complaints are not being answered by the research team.
- You want to talk to someone besides the research team.
- You have questions about your rights as a research subject.
- You want to get more information or provide input about this research.

You may also submit a report to the OHSU Integrity Hotline online at https://secure.ethicspoint.com/domain/media/en/gui/18915/index.html or by calling toll-free (877) 733-8313 (anonymous and available 24 hours a day, 7 days a week).

DO I HAVE TO TAKE PART IN THIS STUDY?

You do not have to join this or any research study. If you do join, and later change your mind, you may quit at any time. If you refuse to join or withdraw early from the study, there will be no penalty or loss of any benefits to which you are otherwise entitled.

HOW DO I TELL YOU IF I WANT TO TAKE PART IN THIS STUDY?

Please indicate whether you provide your consent to participate in this screening portion of the study by selecting "Yes, I would like to participate in the study" or "No, I would not like to participate in the study."
